# Value of multidetector computed tomography angiography before bronchial artery embolization in hemoptysis management and early recurrence prediction: a prospective study

**DOI:** 10.1186/s12890-020-01271-y

**Published:** 2020-08-31

**Authors:** Huu Y Le, Van Nam Le, Ngoc Hung Pham, Anh Tuan Phung, Thanh Tung Nguyen, Quyet Do

**Affiliations:** 1 Department of Respiratory Diseases, Military Hospital 103, Hanoi, Vietnam; 2 Division of Interventional Radiology, Military Hospital 103, Hanoi, Vietnam; 3 Department of Infectious Diseases, Military Hospital 103, Hanoi, Vietnam; 4 Department of Epidemiology, Vietnam Military Medical University, Hanoi, Vietnam; 5 Department of Diagnostic Radiology, Military Hospital 103, Hanoi, Vietnam; 6 Department of Medicine, Vietnam Military Medical University, Hanoi, Vietnam

**Keywords:** Hemoptysis, Multidetector computed tomography angiography, Bronchial artery embolization, Early recurrence

## Abstract

**Background:**

Multidetector computed tomography (MDCT) angiography is a useful examination to detect the source of the bleeding in patients with hemoptysis. The aim of the study was to prospectively evaluate the role and clinical efficacy of MDCT angiography before bronchial artery embolization (BAE) for the management of hemoptysis, and to investigate the predictors of early recurrence.

**Methods:**

It is a double-center study which included 57 hemoptysis patients undergoing MDCT angiography prior to BAE from August 2019 to July 2020. A prospective analysis of culprit arteries detected by MDCT angiography allowed an evaluation of the role of this technique. A follow-up was done to assess the efficacy of BAE with preprocedural MDCT angiography and to explore the risk factors of early recurrent hemoptysis.

**Results:**

The accuracy of MDCT angiography in the identification of culprit arteries was as high as 97.5%. The average number of total culprit arteries per patient was 2.75 ± 1.73. Among which, the average numbers of culprit ectopic bronchial arteries (BAs) and non-bronchial systemic arteries (NBSAs) per patient were 0.21 ± 0.41 and 1.04 ± 1.57, respectively. The immediate clinical success rate, total hemoptysis recurrence rate, and early hemoptysis recurrence rate of BAE following MDCT angiography were 94.7, 18.5, 16.7%, respectively. Aspergilloma (HR = 6.63, 95% CI: 1.31–33.60, *p* = 0.022) was associated with an increase in the risk of early recurrence.

**Conclusions:**

MDCT angiography should be performed before BAE for the management of hemoptysis. Aspergilloma was an independent predictor for early recurrence.

## Background

Hemoptysis is a challenging symptom which frequently involves life-threatening conditions, such as malignancies. Therefore, the diagnostic work-up should be as exhaustive as possible. However, the lack of evidence-based guidelines regarding appropriate diagnostic management may lead to inaccurate diagnosis and delayed treatment of the symptom [1]. The common causes include active/reactivated tuberculosis (TB), bronchiectasis, aspergilloma, respiratory infections, and lung cancer [2–5]. ﻿Because the bronchial circulation is the main source of bleeding in massive hemoptysis, ﻿bronchial artery embolization (BAE) is considered as the first-line therapy as well as a bridge to surgery for control of the hemorrhage. Besides, it is also an effective method for patients with chronic recurrent hemoptysis after the failure of maximum medical treatment or contraindication to surgery [6, 7]. Nevertheless, up to 57.5% of patients could experience the recurrent event after BAE, and this rate increases with time [2]. One of the main reasons is the lack of complete exploration for all culprit vessels, particularly ﻿ectopic bronchial arteries (BAs) and non-bronchial systemic arteries (NBSAs) [2, 8].

In patients with hemoptysis, the identification of sources and causes of bleeding with non-invasive methods prior to interventional techniques is essential. To date, there have been many studies reporting that multidetector computed tomography (MDCT) angiography before BAE plays a critical role in the diagnosis of abnormal arteries and the hemoptysis cause [9–15]. Importantly, it effectively identifies origins and courses of ectopic BAs and NBSAs, which should be useful for the management of this disease [8, 9, 16, 17]. In addition, bronchoscopy has been also known as an important procedure in the work-up of hemoptysis of any severity [18]. It is complementary to computed tomography (CT) scan in the diagnosis of hemoptysis etiology and in the localization of the bleeding source. It may be very useful prior to BAE when CT fails to detect the exact bleeding site and may be therapeutic in case of endobronchial lesions.

However, most reports have been retrospective studies, and the evaluation of efficacy of BAE following MDCT angiography on clinical outcomes is limited. Some literature also has uncertainly recommended MDCT angiography as a regular examination in patients undergoing BAE for hemoptysis [19–22]. Therefore, the main aim of this study is to prospectively assess the detection of culprit arteries and the clinical value of MDCT angiography before BAE for the management of hemoptysis. The second purpose is to investigate the predictors of early recurrence. Besides, TB and post-tubercular sequelae are major causes of hemoptysis requiring BAE [2], particularly in such high TB burden countries as Vietnam [23]. For this reason, we also study the clinical and angiographic characteristics of patients with pulmonary TB history.

## Methods

### Patients and study design

A prospective, double-center study, including 57 consecutive patients with hemoptysis who were referred for BAE, was conducted at Military Hospital 103 and National Lung Hospital, two national hospitals in Hanoi, Vietnam between August 2019 and July 2020. The classification of hemoptysis grades include massive hemoptysis (≥ 300 mL/day or respiratory failure, or hemodynamic instability), moderate hemoptysis (100–300 mL/day), and mild hemoptysis (< 100 mL/day) [2]. All patients underwent MDCT angiography before BAE, and a paired comparison between the angiographic results of two techniques was done. Bronchoscopy was also used to diagnose the hemoptysis etiology in some patients. Enrolled patients were classified into two groups based on the history of pulmonary TB to compare the clinical and angiographic characteristics. Also, we prospectively evaluated the technical success rate and clinical outcomes of BAE with preprocedural MDCT angiography. We excluded patients with a previous history of BAE before August 2019. The protocol of this study was approved by the Institutional Review Board of Vietnam Military Medical University (number: 251/2019) and by the local ethics committees of two participating centers. All participants had provided written informed consents for this research.

### MDCT angiography and image analysis

MDCT angiography was performed with a 64-MDCT scanner (SOMATOM Sensation 64; Siemens Medical Solutions, Forchheim, Germany) in 43 patients (120 kV, 320 mAs, rotation time of 0.5 s, 0.75-mm collimation, pitch of 1.5) and with a 16-MDCT scanner (Brilliance 16; Philips Medical Systems, Ohio, USA) in the remaining 14 patients (140 kV, 70–120 mAs, rotation time of 0.5 s, 0.75-mm collimation, pitch of 1.5). All patients were scanned in a supine position from lung tip to diaphragm. Patients received approximately 80 to 100 mL of contrast material (Omnipaque 300 mgI/mL; GE Healthcare, Oslo, Norway) followed by 50 mL of normal saline solution, which was injected intravenously at a rate of 4 mL/s. The automatic bolus triggering software program was performed, with a circular region of interest positioned at the level of the descending thoracic aorta. Triggered data acquisition began at the contrast enhancement level of 100 Hounsfield unit (HU). Series of images were reconstructed at 1-mm section thickness with 0.6-mm increment. All data of MDCT angiography were transferred to a workstation for post-processing. Two independent radiologists (P.A.T and T.N.T, who had more than 15 years of experience in reading MDCT angiography) analysed the CT images. The inconsistent results were discussed with a board of experts for final decisions.

We first evaluated characteristics of culprit vessels (focused on the number, origin, site of the ostium, diameter, and course), including BAs and NBSAs. BAs were divided into two groups: (a) orthotopic BAs originating from the descending aorta between the level of T5 and T6 vertebrae and (b) ectopic BAs from any level of aorta outside levels T5 and T6 vertebrae, or its branches. NBSAs were defined as arteries which enter the parenchymal through the inferior pulmonary ligament or adherent pleura and their courses were not parallel to the bronchi [14]. BAs were considered abnormal when (a) their diameter was ≥ 2 mm, or (b) their courses were tortuous and could be identified to the hilum. NBSAs were considered abnormal if they were dilated and tortuous, within extrapleural fat related to pleural thickening. Secondly, we investigated the other radiological findings as specific lesions, which could be causes of hemoptysis, such as active/reactivated TB, bronchiectasis, aspergilloma, and malignancy. These characteristics integrated with additional tests (e.g. microscopy, bacterial/MTB/fungal culture, and Xpert MTB/RIF assay of sputum/bronchoalveolar lavage samples, serum antibody test, and histopathology test) for the definitive diagnosis. The extent of lung diseases was classified according to the number of lobes with involvement on chest CT, 1 to 3 lobes, or more than 3 lobes.

### BAE procedure

BAE procedure, including arteriography and embolization, was performed with a 5-Fr introducer sheath (Terumo, Japan) through a common femoral artery access, using the Seldinger technique. Because of the vascular map of MDCT angiography before, selective catheterization of culprit BAs and NBSAs was conducted without aortography. 5-Fr curved catheters, including Cobra, Hook, or Simmons, and right coronary artery catheters were used. Coaxial microcatheters (Carnelian 1.8/2.2-Fr; Tokai Medical Products, Japan) were guided by the 0.016/0.018-in. M guidewire (Terumo, Japan). Transcatheter embolization was completed for all abnormal arteries that met one of the following radiological characteristics: (a) tortuous enlargement of BAs and/or NBSAs which provided the region of parenchymal staining, or (b) a shunt into pulmonary vessels [14]. Embolic agents were polyvinyl alcohol (PVA; size range, 350–710 μm; Contour; Boston Scientific, USA) and a combination of PVA and gelatin sponge (Gelfoam; BioSphere Medical, USA). BAE was performed by four interventional radiologists (D.N.B and N.T.T in the first center, and T.N.T and N.D.M in the second center), all of whom have had more than ten years of experience in vascular intervention.

We recorded the number, origins, and courses of culprit BAs and NBSAs. They were considered as gold standards for diagnosis and compared to the results of MDCT angiography. Also, the rate of technical success was noted. It was defined as the embolization of all observed abnormal arteries.

### Follow-up and clinical outcome

After BAE, all participating patients were followed up with a regular re-examination or by telephone to assess the clinical outcomes of BAE with preprocedural MDCT angiography. The first outcome was the rate of immediate clinical success, of which the bleeding was stopped or significantly reduced within 24 h of BAE. The second outcome was the rates of hemoptysis recurrence, which referred to significant hemoptysis occurring after discharge. Among these, early recurrence appeared within 3 months of BAE [2].

### Statistical analysis

Data of continuous variables were presented as mean ± SD [standard deviation] and that of categorical variables were presented as numbers and percentages. The baseline characteristics between history and non-history of pulmonary TB groups were compared by using *t*-test for continuous variables, and *χ* ^*2*^ test and Fisher’s exact test for categorical variables. The average numbers of culprit arteries per patient between two groups were analysed by the Mann-Whitney *U* test. To explore the risk factors associated with early recurrence, univariate and multivariate Cox proportional hazards regression models were performed. A *p*-value of less than 0.05 was considered as the statistical significance. All analyses were performed with SPSS version 25.0 (IBM, Armonk, NY, USA) statistical software.

## Results

### Baseline characteristics

The study included 57 patients from August 2019 to July 2020. Among whom, there were 40 males (70.2%), and the mean ± SD age at admission was 54.4 ± 16.6 years (range, 18–86 years). The incidences of cardiovascular diseases, diabetes mellitus, and chronic liver diseases were 19.3% (11/57), 8.8% (5/57), 7.0% (4/57), respectively. It is noted that the top three common causes of hemoptysis requiring BAE were bronchiectasis (*n* = 23, 40.4%), active/reactivated TB (*n* = 22, 38.6%), and aspergilloma (*n* = 10, 17.5%). Notably, of 57 patients, 23 (40.4%) had a history of pulmonary TB, and 21 (36.8%) had massive hemoptysis. The mean ± SD duration from BAE to death or the end day of follow-up was 4.7 ± 3.2 (range, 1–12 months). The baseline characteristics of the participants are reported in Table 1.

**Table 1:** Demographic and clinical characteristics

Characteristics	*N* = 57
**Age, years**	54.4 ± 16.6
**Sex**	
Male	40 (70.2)
Female	17 (29.8)
**Underlying diseases**	
Cardiovascular diseases	11 (19.3)
Diabetes mellitus	5 (8.8)
Chronic liver diseases	4 (7.0)
**Pulmonary TB history**	23 (40.4)
**Extent of lung diseases**	
≤ 3 lobes	31 (54.4)
> 3 lobes	26 (45.6)
**Grade of hemoptysis**	
Massive	21 (36.8)
Moderate	18 (31.6)
Mild	18 (31.6)
**Etiology**	
Bronchiectasis	23 (40.4)
Active/reactivated TB	22 (38.6)
Aspergilloma	10 (17.5)
Others^†^	2 (3.5)
**Embolic agent**	
Polyvinyl alcohol	43 (75.4)
Combination^*^	14 (24.6)
**Follow-up duration, months**	4.7 ± 3.2

Data are reported as n (%) or mean ± SD [standard deviation]

^†^Other etiologies include nontuberculous Mycobacteria (NTM, *n* = 1) and unknown cause (n = 1)

^*^Combination of polyvinyl alcohol and gelatin sponge

*TB:* tuberculosis

### MDCT angiography and BAE procedure for detection of culprit arteries

One hundred fifty-seven vessels were detected as abnormal arteries by MDCT angiography, of which two orthotopic BAs were diagnosed as normal vessels by BAE. There were also 157 vessels identified as culprit arteries by BAE procedure. Among which, one NBSA originating from a subclavian artery and the other from an intercostal artery were missed on MDCT angiography. Out of a total of 159 arteries, 155 were reasonable concordance between the results of two techniques. Therefore, the accuracy of MDCT angiography in the detection of culprit arteries was 97.5% (155/159). The average number of culprit orthotopic BAs, ectopic BAs, and NBSAs per patient were 1.54 ± 0.73, 0.21 ± 0.41, 1.04 ± 1.57, respectively. Totally, the average number of culprit arteries per patient was 2.75 ± 1.73 (Table 2).

**Table 2:** Detection of culprit arteries of MDCT angiography and BAE procedure

Culprit arteries	Number of culprit arteries		Number of culprit arteries per patient
	MDCT angiography	BAE procedure	
**Orthotopic BAs**			
Left bronchial	25	25	
Right bronchial	12	11	
Common bronchial	15	15	
RIBT	36	35	
Total	88	86	1.54 ± 0.73
**Ectopic BAs**			
Aortic arch	8	8	
Subclavian or its branches	2	2	
Internal mammary	2	2	
Total	12	12	0.21 ± 0.41
**NBSAs**			
Intercostal	29	30	
Subclavian or its branches	13	14	
Internal mammary	12	12	
Thyrocervical trunk	2	2	
Inferior phrenic	1	1	
Total	57	59	1.04 ± 1.57
**Total**	157	157	2.75 ± 1.73

Data are reported as n or mean ± SD [standard deviation]

*MDCT:* multidetector computed tomography, *BAE:* bronchial artery embolization, *RIBT:* right intercostobronchial trunk, *BAs:* bronchial arteries, *NBSAs:* non-bronchial systemic arteries

**Fig. 1:**
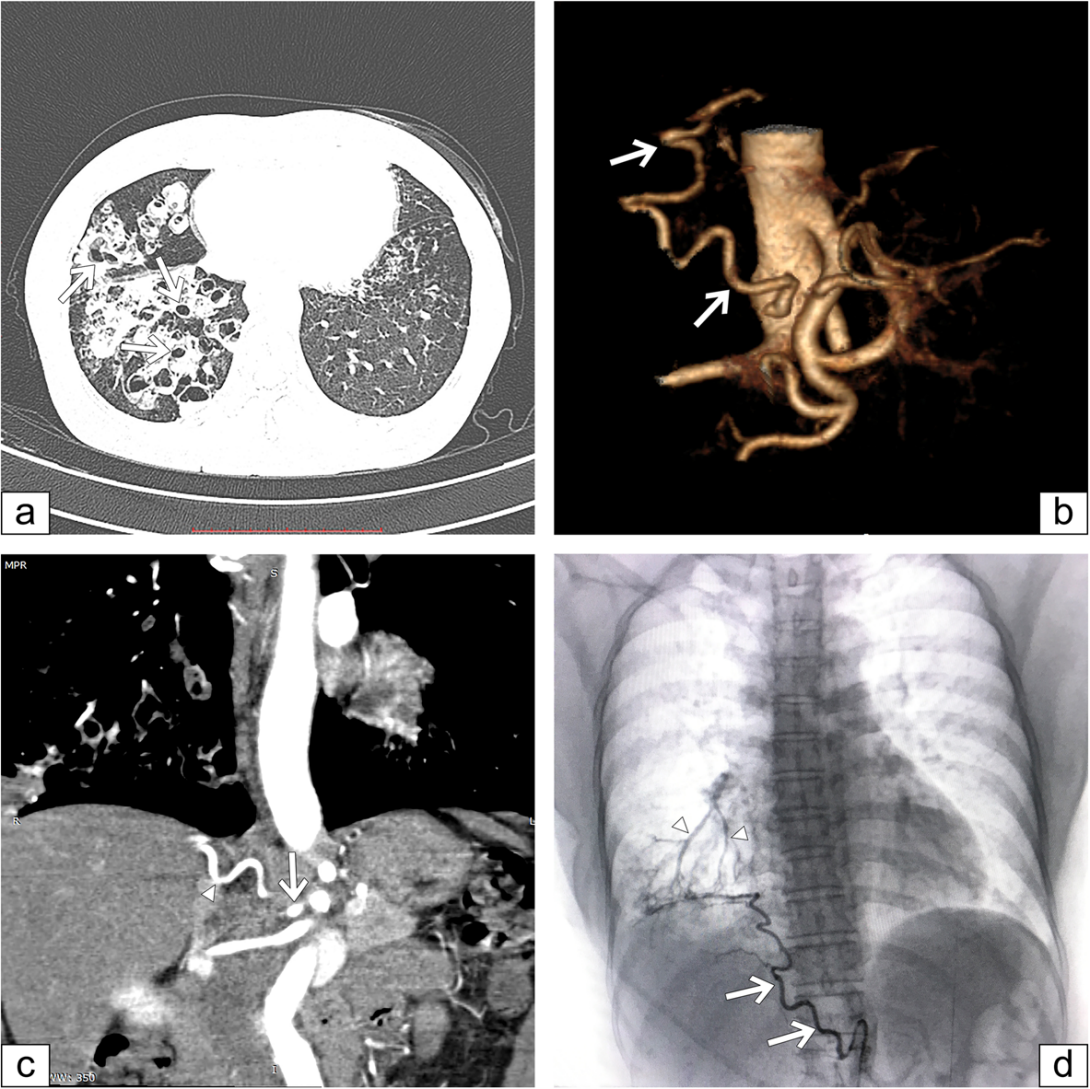
A case presentation with moderate hemoptysis. **a** A chest computed tomography scan demonstrates multifocal bronchiectasis that involves the middle and lower lobes of the right lung *(arrows).* **b** Volume rendered image shows hypertrophy and with a tortuosity of a right inferior phrenic artery *(arrows).* **c** Coronal maximum intensity projection shows the origin *(arrow)* and part of the course *(arrowhead)* of a right inferior phrenic artery. **d** The selective arteriogram of this artery confirms the presence of abnormal ﻿enlargement with a tortuous course *(arrows)* and shunts into pulmonary vessels *(arrowheads)*

Figure 1 showed angiographic results of a 50-year-old female with persistent hemoptysis caused by bronchiectasis. A culprit right inferior phrenic artery which originated from the abdominal aorta supplied to the base of the right lung. It was detected by both MDCT angiography and BAE procedure.

### Technical and clinical outcomes of BAE with preprocedural MDCT angiography

Among enrolled patients received BAE, the technical failure occurred in 4 patients due to artery tortuosity (*n* = 1), ostial narrowing (n = 1), and acute or multiple branching (*n* = 2). As a result, the rate of technical success was 93.0% (53/57). Regarding the clinical outcomes, the rate of immediate clinical success was 94.7% (54/57). Of the 3 patients who experienced significant hemoptysis within 24 h of BAE, one underwent an urgent lobectomy and two continuously received the medical treatment. In patients with immediate clinical success, 10/54 (18.5%) were recurrent bleeding with the follow-up periods ranging from 1 to 12 months. The rate of early recurrence was 16.7% (9/54), and there were 4 deaths because of massive hemoptysis recurrence (*n* = 3) and severe underlying lung diseases (*n* = 1).

### Predictors of early recurrence

We excluded three patients without immediate clinical success and two patients in the other etiology group, the remaining 52 patients were analysed to explore the risk factors of early recurrence. Table 3 provides an overview of all variables that could be predictors. By using univariate Cox regression analysis, we initially evaluated the variable that presented a significant difference (*p* < 0.05). The results revealed that aspergilloma (HR [hazard ratio] = 5.25, 95% CI: 1.16–23.73, *p* = 0.031) was associated with the early recurrence. Multivariate analysis of three variables, including the etiology of hemoptysis, extent of lung diseases, and grade of hemoptysis, was performed. Our findings also indicated that aspergilloma (HR = 6.63, *p* = 0.022) was the risk factor of early recurrence for patients who received BAE with preprocedural MDCT angiography.

**Table 3:** Univariate and multivariate Cox regression analyses of risk factors for early recurrence after BAE following MDCT angiography

Variable	Univariate analysis		Multivariate analysis	
	HR (95% CI)	***p***-value	HR (95% CI)	***p***-value
**Age, years**				
≤ 50	3.48 (0.83–14.59)	0.088		
> 50	1			
**Sex**				
Male	3.07 (0.38–24.96)	0.294		
Female	1			
**Cardiovascular diseases**	0.50 (0.06–4.07)	0.517		
**Diabetes mellitus**	1.57 (0.19–12.80)	0.671		
**Chronic liver diseases**	3.94 (0.79–19.53)	0.093		
**Pulmonary TB history**	2.49 (0.60–10.43)	0.212		
**Extent of lung diseases**				
> 3 lobes	0.67 (0.16–2.81)	0.584	0.45 (0.10–2.09)	0.308
≤ 3 lobes	1		1	
**Grade of hemoptysis**		0.262		0.264
Massive	2.47 (0.48–12.75)	0.280	3.06 (0.50–18.74)	0.227
Moderate	0.51 (0.05–5.64)	0.582	0.60 (0.05–6.89)	0.684
Mild	1		1	
**Etiology**		0.016		0.019
Aspergilloma	5.25 (1.16–23.73)	0.031	6.63 (1.31–33.60)	0.022
Bronchiectasis	0.34 (0.04–3.23)	0.344	0.54 (0.50–5.85)	0.612
Active/reactivated TB	1		1	
**Embolic agent**				
Polyvinyl alcohol	2.26 (0.28–18.37)	0.446		
Combination^†^	1			

^†^Combination of polyvinyl alcohol and gelatin sponge

*TB:* tuberculosis, *HR:* hazard ratio

### Comparison of groups

Of all 57 patients, there were 23 (40.4%) with and 34 (59.6%) without pulmonary TB history (Table 4). A comparison of two groups showed no significant differences in sex, mean age, the extent of lung diseases, grade of hemoptysis, technical success, immediate clinical success, and recurrence with the same follow-up durations (all *p* > 0.05). However, it is noted that the number of total culprit arteries and culprit NBSAs was significantly higher in the group of pulmonary TB history (3.26 ± 1.86 vs. 2.41 ± 1.56, *p* = 0.031, and 1.61 ± 1.83 vs. 0.65 ± 1.25, *p* = 0.008, respectively). In contrast, the number of culprit orthotopic BAs and ectopic BAs was similar between two groups (*p* = 0.305 and *p* = 0.157, respectively).

**Table 4:** Comparison between history and non-history of pulmonary tuberculosis groups

	History of pulmonary tuberculosis group (n = 23)	Non-history of pulmonary tuberculosis group (***n*** = 34)	***p***-value
**Male sex**	17 (73.9)	23 (67.6)	0.612
**Age, years**	55.0 ± 16.3	54.0 ± 17.0	0.813
**Extent of lung diseases**			0.783
≤ 3 lobes	12 (52.5)	19 (59.9)	
> 3 lobes	11 (47.8)	15 (44.1)	
**Grade of hemoptysis**			0.911
Massive	8 (34.8)	13 (38.2)	
Moderate	7 (30.4)	11 (32.4)	
Mild	8 (34.8)	10 (29.4)	
**Number of culprit artery**			
Orthotopic BA	1.35 ± 0.65	1.62 ± 0.74	0.305
Ectopic BA	0.30 ± 0.47	0.15 ± 0.36	0.157
NBSA	1.61 ± 1.83	0.65 ± 1.25	0.008
Total	3.26 ± 1.86	2.41 ± 1.56	0.031
**Technical success**	21 (91.3)	32 (94.1)	1.000
**Immediate clinical success**	22 (95.7)	32 (94.1)	1.000
**Recurrence**^†^	7 (30.4)	6 (17.6)	0.259
**Follow-up duration, months**	5.2 ± 3.0	4.4 ± 3.4	0.211

Data are reported as mean ± SD [standard deviation] or n (%)

*BA:* bronchial artery, *NBSA:* non-bronchial systemic artery

^†^Including recurrence in immediate periods and during follow-up duration

## Discussion

This study prospectively investigated the clinical value of MDCT angiography before BAE in 57 patients for the management of hemoptysis. Also, the study was to explore the risk factors of early recurrence and the clinical and angiographic characteristics of patients with pulmonary TB history. The common causes of hemoptysis in this research were bronchiectasis, TB, and aspergilloma. Our findings showed some differences in comparison with other articles as mentioned above [3, 4]. They reported that lung cancer and respiratory infections were more frequent than other etiologies. However, bronchiectasis and TB were also the main causes of bleeding in several reports [2, 5]. The accuracy of MDCT angiography in the detection of culprit arteries was high (97.5%), and the efficacy of BAE with ﻿a corresponding preprocedure was acceptable. Aspergilloma was associated with an increase in the risk of early hemoptysis recurrence. Among hemoptysis patients requiring BAE, many patients (23/57, 40.4%) had a history of pulmonary TB. There were no important differences between two groups classified according to pulmonary TB history, excluding the average number of total culprit arteries and culprit NBSAs per patient.

Since the first introduction in 1974 by Remy et al. [24], BAE has been known as an effective option for the control of massive and recurrent hemoptysis until now [6, 7, 25, 26]. Identification of the source and site of the bleeding before BAE is critical to completely search for all abnormal vessels, which can improve the procedural efficiency. In the past decades, a study by Furuse et al. was designed to determine the effect of CT scan on the visibility of BAs and the depiction of its courses [10]. However, the resolution is not high enough to identify culprit BAs exactly. Based on the development of image quality, several reports have shown that MDCT angiography adequately provides the detection and depiction of both abnormal BAs and NBSAs [11, 14–17, 27–29]. Our findings seem to be consistent with Li et al. (2019) who found that the rate of agreement between MDCT angiography and BAE in the diagnosis of arterial abnormalities was as high as 98.8% [17]. Notably, the average number of culprit ectopic BAs and NBSAs per patient in current study is even higher than those of their results.

As mentioned in the literature review, the clinical outcomes of BAE were vastly different among studies. It is presented that the immediate clinical success rate ranges from 70 to 99%, and the hemoptysis recurrence rate ranges from 9.8 to 57.5% [2]. MDCT was done in several studies, but none of these authors used MDCT angiography for the delineation of BAs and NBSAs [30–37]. In 2019, Li et al. performed the first investigation with a control group to assess the clinical effect of BAE with preprocedural MDCT angiography for the management of hemoptysis [17]. Their study confirmed that a higher rate of immediate clinical success (97.2% vs. 88.2%) was achieved in group with MDCT angiography before BAE. As compared to the control group, it can also be useful to reduce the risk of recurrent hemoptysis (11.7% vs. 20.0%), especially early recurrence (3.9% vs. 13.3%). Another report has shown that the clinical success rate during follow-up (1–14 months) was obtained in 94% (50/53) of patients who successfully underwent BAE following MDCT angiography [28]. The findings of the current study are not as good as the previous ones, but they are acceptable. A possible explanation for these results may be the small sample size and differences in the technical success and embolic agents. Despite potential benefits, the existing body of literature that recommends MDCT angiography as a regular examination prior to BAE remains scarce. The reason for this is not clear but it may be due to lack of data on clinical outcomes of this procedure. According to the American College of Radiology and Spanish Society of Pneumology and Thoracic Surgery (SEPAR) guidelines, this technique should be conducted in patients who experience significant hemoptysis but the strength of recommendation is low [21, 22]. Until now, it is often performed by an individualized decision based on institutional availability.

Surprisingly, aspergilloma was found as a predictor for early recurrence among hemoptysis patients treated by BAE following MDCT angiography. A recent systematic review by Panda et al. (2017) summarized that technical failure due to missing culprit vessels or embolization ﻿inability might be causes of early recurrence [2]. However, the high rate of recurrence for patients with aspergilloma has been presented in some published articles [25, 37–41]. Hwang et al. (2013) and van den Heuvel et al. (2007) reported that aspergilloma was significantly associated with re-bleeding after BAE (OR [odds ratio] = 3.557, *p* = 0.003 and OR = 5.1, *p* < 0.05, respectively) [38, 41]. About pathophysiology, triggered by hypoxia, vasculitis, and architectural distortion, there is an opening up of bronchial arterial and pulmonary arterial anastomotic plexus, then becoming targets of erosion and hemoptysis. It is shown that the hemorrhagic sources are often bronchial arteries and can be secondary from a variety of origin, precluding a complete devascularization [42]. Therefore, wedge resection should be considered as a definitive treatment in massive or recurrent hemoptysis patients who can be operable [42]. Cavernostomy and thoracoplasty were also determined the safety and efficacy for high-risk patients with aspergilloma [43].

The third question in this research was what the similarities and differences between hemoptysis patients with and without pulmonary TB history were, who received BAE following MDCT angiography. Consistent with the literature [38, 41, 44], this study found that the incidence of a pulmonary TB history was a rather high rate (40.4%). Two groups were similar in sex, mean age, the extent of lung diseases, grade of hemoptysis, and technical success and immediate clinical success rate. Regarding angiographic findings, our data support evidence from previous observations (e.g. Lee et al., 2007) that patients with pulmonary TB history have a significantly higher number of total culprit arteries and culprit NBSAs than patients without pulmonary TB history [44]. According to prior studies, hemoptysis recurrence after BAE tended to be higher in patients with TB (chronic or active/reactivated TB) [37, 41, 44, 45]. These outcomes are contrary to the current study, which has reported that pulmonary TB history was not related to re-bleeding. This difference may partly be explained by a small sample size and/or more quality of the treatment, or that we were concentrated on pulmonary TB history patients, not on active TB.

This study has several limitations. Firstly, it has no control group. Therefore, we have compared with previous studies to evaluate the value of MDCT angiography before BAE. But randomised controlled trials will be recommended to gain an insightful picture of this technique. Secondly, only short-term outcomes were covered in the scope of this study. However, it should be noted that the recurrent event could occur from 6 months to 1 year after BAE [2]. Hence, further studies are needed to have a more comprehensive view on the efficacy of BAE with preprocedural MDCT angiography. Finally, the number of patients with aspergilloma is small (*n* = 10), and this might be a cause of bias in our results. In addition, the incidence of hemoptysis recurrence might vary by many factors, these findings cannot be extrapolated to all patients.

## Conclusions

MDCT angiography could be considered as a regular examination prior to BAE for the management of hemoptysis. Aspergilloma was a risk factor for early recurrence. Patients with pulmonary TB history have a significantly higher number of total culprit arteries and culprit NBSAs than patients without pulmonary TB history.

## Data Availability

The datasets used and/or analysed during the current study are available from the corresponding author upon reasonable request.

## Abbreviations

BAs: bronchial arteries
BAE: bronchial artery embolization
MDCT: multidetector computed tomography
MTB: *Mycobacterium tuberculosis*
NBSAs: non-bronchial systemic arteries
TB: tuberculosis
